# Repetitive Transcranial Direct Current Stimulation Induced Excitability Changes of Primary Visual Cortex and Visual Learning Effects—A Pilot Study

**DOI:** 10.3389/fnbeh.2016.00116

**Published:** 2016-06-03

**Authors:** Matthias Sczesny-Kaiser, Katharina Beckhaus, Hubert R. Dinse, Peter Schwenkreis, Martin Tegenthoff, Oliver Höffken

**Affiliations:** ^1^Department of Neurology, BG-Universitaetsklinikum BergmannsheilBochum, Germany; ^2^Institute of Neuroinformatics, Ruhr-University BochumBochum, Germany

**Keywords:** non-invasive brain stimulation, transcranial direct current stimulation, primary visual cortex, cortical excitability, perceptual learning, humans

## Abstract

Studies on noninvasive motor cortex stimulation and motor learning demonstrated cortical excitability as a marker for a learning effect. Transcranial direct current stimulation (tDCS) is a non-invasive tool to modulate cortical excitability. It is as yet unknown how tDCS-induced excitability changes and perceptual learning in visual cortex correlate. Our study aimed to examine the influence of tDCS on visual perceptual learning in healthy humans. Additionally, we measured excitability in primary visual cortex (V1). We hypothesized that anodal tDCS would improve and cathodal tDCS would have minor or no effects on visual learning. Anodal, cathodal or sham tDCS were applied over V1 in a randomized, double-blinded design over four consecutive days (*n* = 30). During 20 min of tDCS, subjects had to learn a visual orientation-discrimination task (ODT). Excitability parameters were measured by analyzing paired-stimulation behavior of visual-evoked potentials (ps-VEP) and by measuring phosphene thresholds (PTs) before and after the stimulation period of 4 days. Compared with sham-tDCS, anodal tDCS led to an improvement of visual discrimination learning (*p* < 0.003). We found reduced PTs and increased ps-VEP ratios indicating increased cortical excitability after anodal tDCS (PT: *p* = 0.002, ps-VEP: *p* = 0.003). Correlation analysis within the anodal tDCS group revealed no significant correlation between PTs and learning effect. For cathodal tDCS, no significant effects on learning or on excitability could be seen. Our results showed that anodal tDCS over V1 resulted in improved visual perceptual learning and increased cortical excitability. tDCS is a promising tool to alter V1 excitability and, hence, perceptual visual learning.

## Introduction

Transcranial direct current stimulation (tDCS) is a non-invasive tool to modulate cortical excitability in a polarity dependent manner. The first human tDCS studies focused on the primary motor cortex (M1; Nitsche and Paulus, 2000, 2001; Nitsche et al., 2003a; Lang et al., 2004). Here, anodal tDCS applied over motor cortex resulted in intracortical facilitation, cathodal tDCS in intracortical inhibition assessed by transcranial magnetic stimulation. Different physical parameters influence the efficacy of tDCS and its after-effects, such as current strength, current density, and stimulation duration: the stronger and the longer the stimulation duration, the stronger the after-effects (Nitsche et al., 2008). However, there are several limitations. Complex homeostatic plastic mechanisms limit uncontrolled increase in synaptic effectiveness and prevent potential destabilization of the neuronal system (Bienenstock et al., 1982; Fricke et al., 2011). In this concept, the activation history of the postsynaptic neuron decides whether a facilitating tDCS protocol leads to further facitilation or even inhibition (Lang et al., 2004; Fricke et al., 2011). Another factor that influences the duration of tDCS-induced offline effects is a repetition of stimulation over several consecutive days, which can activate LTP-like molecular mechanisms (Fritsch et al., 2010).

In the visual system polarity-specific tDCS effects could be demonstrated, too. Anodal tDCS, applied over the visual cortex (V1), decreased phosphene thresholds (PTs), which indicated an enhancement of cortical excitability, whereas, cathodal tDCS increased thresholds. These effects could be observed for moving and for stationary phosphenes (Antal et al., 2003a,b). Offline effects depended on the duration of the stimulation period. Effects during stimulation were more consistent than the after-effects (Accornero et al., 2007).

Not only tDCS-induced alterations of electrophysiological parameters are of interest, but also tDCS-induced changes of visual learning. Performance of a visuomotor task was improved during anodal tDCS over extrastriatal visual cortical area V5 or M1, whereas cathodal stimulation had no significant effect (Antal et al., 2004). Further studies investigated manipulation of visual abilities. Here, anodal tDCS over V1 improved significantly contrast sensitivity and detection sensitivity, while cathodal tDCS showed no effect (Kraft et al., 2010; Olma et al., 2011). But, like in the motor system, some tDCS-studies on visual learning revealed diverging effects. Fertonani et al., 2011 and Pirulli et al., 2013 could not prove tDCS-effects (anodal or cathodal) on visual learning when tDCS was applied once before or during discrimination task. To analyze the mechanisms of neuronal plasticity in V1, patients with hemianopia due to stroke received visual restoration therapy and anodal tDCS (Plow et al., 2012). In spite of the small sample size (*n* = 8), the authors found beneficial effects of a combination of both therapies. This indicates that anodal tDCS can also improve neuronal plasticity in stroke and altered neuronal tissue by increasing excitability and inducing cortical remapping.

Taken together, these studies demonstrated significant tDCS-effects on visual learning. A current debate concerns the question in which functional systems anodal and cathodal tDCS actually have polarity-specific, that is opposing, effects. Some studies even showed functional improvement after cathodal tDCS (Dockery et al., 2009; Elmer et al., 2009; Berryhill et al., 2010; Williams et al., 2010) while anodal tDCS had no significant or only minor effects. In Williams’s study, cathodal tDCS yielded improvement of motor functions by reducing inhibitory influences of the contralateral hemisphere (Williams et al., 2010). The mechanism leading to improved cognitive functioning after cathodal tDCS remain unclear (Dockery et al., 2009; Elmer et al., 2009; Berryhill et al., 2010). Furthermore, depending on the time of stimulation, anodal tDCS can decrease learning performance (Stagg et al., 2011). In a sham-controlled tDCS study, Peters et al. (2013) showed that anodal tDCS even blocked the consolidation of visual performance learning in a contrast detection task. Together, these results show that anodal-cathodal stimulation effects on learning and behavior cannot be categorized easily. Therefore, when choosing a study design, it is important to include all stimulation types (cathodal, anodal and sham) to explicitly analyze and interpret different effects.

Up to now, no visual learning study investigated induced changes in cortical excitability or the correlation between cortical excitability and visual learning. Correlation analyses between both parameters might provide an insight into underlying mechanisms of visual perceptual learning. For the primary somatosensory cortex (S1), electrophysiological measurements or functional magnetic resonance imaging revealed improved perceptual learning and changes in excitability or cortical activity after high frequency transcranial magnetic stimulation (TMS) over S1 (Tegenthoff et al., 2005; Ragert et al., 2008). Both parameters did not correlate significantly, but were positively associated: the higher the cortical excitability, the greater the learning effect. The authors concluded that the observed improvement was probably based on processes that involve increased cortical excitability. Studies investigating the link between cortical excitability in V1 and perceptual learning have not been published so far.

Since in our study, tDCS was applied over V1, it was important to choose a learning paradigm and excitability parameters targeted specifically at this region. So, we used PTs and paired-stimulation visually evoked potentials (psVEPs). Although it is conceivable that both methods target aspects of visual cortex excitability, they may be mediated through different underlying mechanisms (Höffken et al., 2013). Whereas phosphenes are supposed to be generated not only in V1 but also in extrastriatal cortical areas (Kammer et al., 2001), VEPs arise primarily from V1 (Di Russo et al., 2005). To assess visual perceptual learning, we used an orientation-discrimination task (ODT). Schoups et al. (2001) demonstrated that the psychophysiological learning effect in an ODT is linked with neuronal performance of specialized cortical neurons in V1.

In summary, the aim of our present study was to investigate the impact of anodal and cathodal tDCS applied over V1 for four consecutive days upon visual perceptual learning; as well as its influence on cortical excitability, measured by PTs and psVEPs. We hypothesized that anodal tDCS would decrease paired-stimulation suppression of VEPs and PT, and improve discrimination learning. In contrast, cathodal tDCS was supposed to reduce cortical excitability but to have no or only minor effects on visual learning. Furthermore, we postulated a significant correlation between excitability and learning effect.

## Materials and Methods

### Participants

We collected and analyzed data of 30 healthy subjects (15 males and 15 females, mean age and SD: 24.7 ± 2.8 years). Subjects were randomly assigned to three equally-sized groups (*n* = 10) as follows: cathodal tDCS group (5 males and 5 females; 25.5 ± 3.1 years), anodal tDCS group (5 males and 5 females; 25.1 ± 3.3 years) and sham tDCS group (5 males and 5 females; 23.6 ± 1.5 years). Participants did not take any regular medication and did not suffer from neurological diseases or psychiatric disorders, nor from any kind of headache, and had no metallic implants. All participants had normal or corrected to normal vision and wore their corrective eyeglasses during testing. All individuals participating in the study gave their informed consent. Relevant safety procedures for tDCS were adhered to Nitsche et al. (2003b) and Poreisz et al. (2007)). The study was approved by the Ethics Committee of the Ruhr-University Bochum (register no. 4300-12) and was performed in accordance with the Declaration of Helsinki.

### Experimental Design

**Figure 1:**
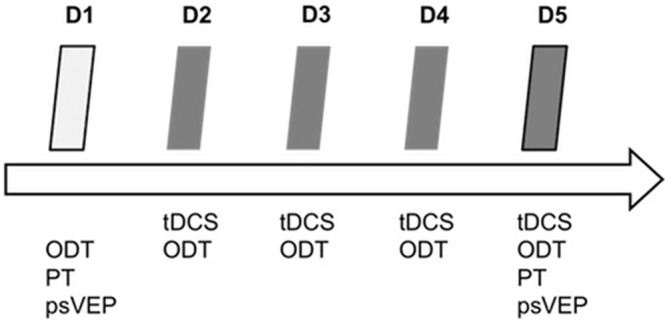
**Experimental design.** tDCS, transcranial direct current stimulation; ODT, orientation discrimination task; PT, phosphene threshold; psVEP, paired-stimulation visual evoked potentials; D1–D5, day 1–day 5.

We performed baseline measurements (PT, orientation discrimination and psVEPs) on day 1 (D1). On the following four consecutive days (D2 to D5), the participants had to learn the visual ODT while receiving tDCS for 20 min. After tDCS and learning task on day 5 (D5), we additionally assessed excitability parameters (PT and psVEPs). Figure 1 shows the experimental design.

### Transcranial Direct Current Stimulation

tDCS was delivered by a battery-driven constant DC current stimulator (NeuroConn, Ilmenau, Germany) using a pair of rubber electrodes in a 5 cm × 7 cm (surface 35 cm^2^) 0.9% saline-soaked synthetic sponge. The electrodes were placed according to the International 10–20-system (American Clinical Neurophysiology Society, 2006). The stimulation electrode was placed over O_Z_, whereas the reference electrode was positioned over C_Z_. The subjects were blind with regard to the type of stimulation (anodal, cathodal or sham). To ensure a double-blind procedure, the experimenter received a 6-digit number from the main investigator that encoded the type of stimulation for a given subject (so called “study mode” of the NeuroConn tDCS device). So, neither the experimenter nor the subject was aware of the type of DC stimulation (anodal, cathodal or sham). The current was applied for approximately 20 min (1170 s) with an intensity of 1.0 mA (current density 0.029 mA/cm^2^, total charge 0.33 C/cm^2^). An ampere meter integrated in the DC stimulator controlled constant current flow. Actual voltage, current and impedance were shown on the display and could be controlled by the experimenter. Using a ramp-like switch, current strength of tDCS gradually increased for the first and decreased for the last 15 s. During the sham condition current flowed for a period of 30 s at the beginning of stimulation and was then turned off. This procedure induces a weak-prickling sensation, making it impossible for a subject to distinguish the stimulation conditions. The stimulation was repeated daily for four consecutive days.

### Visual Evoked Potentials

**Figure 2:**
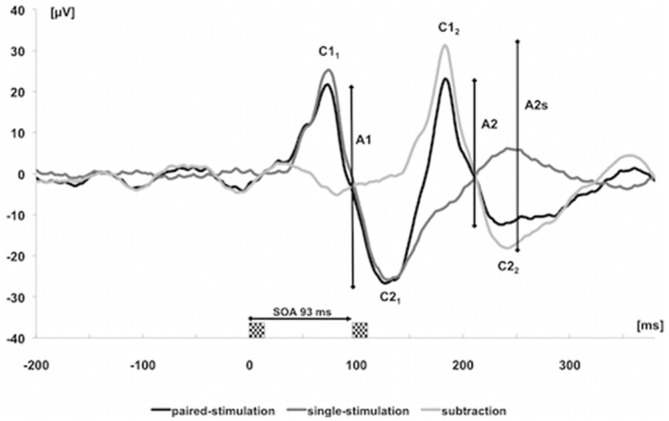
**Visually evoked potentials (VEPs) over cortical Oz of one subject after single (dark gray trace) and paired stimulation with stimulation onset asynchrony (SOA) of 93 ms (black trace).** The label C was used to characterize the positive and negative components of the first and second response. The light gray trace results by subtracting the single-stimulation trace from the paired-stimulation trace. The analyzed amplitudes of the first response (A1 = C2_1_–C1_1_) and second response (A2 = C2_2_–C1_2_) after paired stimulation are marked by vertical bars; amplitudes of the second response after subtracting the response to a single stimulation are labeled as amplitudes of the second (A2s).

Subjects were seated in a darkened room, at a distance of 50 cm from a screen (cathode ray tube, frame rate 75 Hz, pixel resolution 800 × 600, spanning 23° × 17° of visual angle). They were instructed to relax and to concentrate on a small dim fixation mark in the center of the display during the entire measurement. The stimuli of the VEPs were generated by means of the EP2000-System (Bach, 2000). We recorded the potentials with a 32-channel-amplifier (Brain Amp, Brain Products, Germany, sampling rate 5 kHz, band-pass filtering between 2 and 1000 Hz) and stored them for offline analyses. The paired-stimulation paradigm consisted of an onset-offset checkerboard pattern with 36% contrast and a check size of 0.5° with a mean luminance of 16 cd/m^2^. To examine paired-stimulation inhibition, we used a stimulation onset asynchrony (SOA) of 93 ms, which revealed reliable paired-stimulation inhibition in recent studies (Höffken et al., 2008, 2009). The stimuli were presented in frames of 13.33 ms corresponding to the frame rate of the tube. After the first checkerboard stimulus, a homogenous gray background without a change in mean luminance appeared for six frames (80 ms). Subsequently the second checkerboard stimulus followed for one frame. The trials containing these paired stimuli were separated by an intertrial interval of 1000 ms resulting in a frequency of about 1 Hz. Ten trials with paired stimuli were followed by 10 trials with single stimuli, with identical contrast and luminance as before, constituting one cycle. Altogether, the stimulation paradigm consisted of four cycles of 10 single and 10 paired stimuli each. Evoked potentials after single and paired stimulation were recorded in epochs from 200 ms before and 400 ms after the stimulus, baseline corrected to the pre-stimulus and averaged. Signals exceeding 140 μV were rejected as artifacts and not counted for the stimulation sequence. The positive peak occurring earlier than 100 ms after stimulus onset, was labeled C1 and the negative peak, occurring later than 100 ms after stimulus onset, was labeled C2 (Odom et al., 2010). Considering ppVEP, the amplitudes between C1 and C2 were named A1 (first amplitude) and A2 (second amplitude). We subtracted the response of the single stimulation from the response of the paired-stimulation (A2s) to remove confounds from superposition. Paired-stimulation was expressed as a ratio (A2s/A1) of the amplitudes of the second (A2s) and first (A1) stimulus (Höffken et al., 2013; Figure 2).

### Phosphene Threshold

Subjects were requested to fixate a crosshairs in front of them while seated in a semi-darkened room with their head fixed on a chinrest. Single biphasic TMS pulses were administered using a figure-of-eight shaped coil with the handle orientated upwards, attached on a tripod and placed 1–5 cm above the inion (coil: outside diameter 8.7 cm, peak magnetic field strength 2.2 T, peak electric field strength 660 V/m) using a Magstim Rapid stimulator (Magstim, Whitland, Dyfed, UK). First, we tried to generate phosphenes by starting with 80% of maximal stimulator output and raising the output in increments of 5% until a stable phosphene was perceived. Secondly, we determined the PT (Sparing et al., 2002). We started with 30% and increased the output in 5%-steps until phosphenes were reported. To confirm and refine the PT, we increased and decreased the output in a randomized order around the supposed threshold. The exact PT was defined as the minimum stimulus intensity of stimulator output able to evoke phosphene perception in at least three of five repetitions at the same output.

### Orientation Discrimination Task

**Figure 3:**
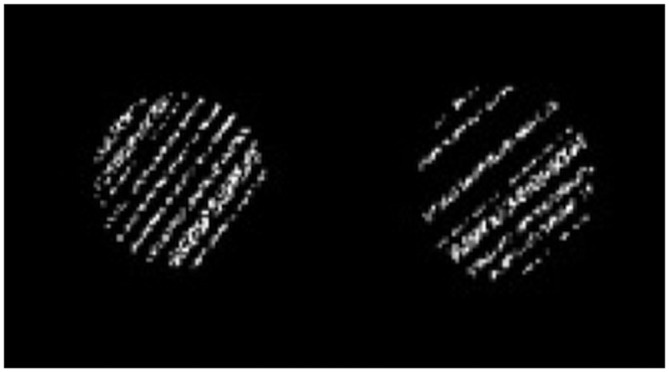
**Orientation discrimination task (ODT).** Two random examples are shown. On the left, an example with 7° counterclockwise tilt relative to the reference oblique orientation is shown. On the right, the bars tilted clockwise to the reference orientation.

The subjects sat in a semi-darkened room in front of a screen in a distance of 50 cm (pixel resolution 1024 × 768, illuminance 3.7 Lux (± 0.1 Lux)). A computer-generated circular stimulus (diameter 2.5 cm, contrast 80%, luminance 25 cd/m^2^), consisting of light and dark bars that formed a noise field, was presented in the center of the black screen. The light bars consisted of white and black pixels (ratio 1:1) in a random order, whereas black bars contained only black pixels. The stimulus was shown for 300 ms and subjects had to respond within the next 700 ms. Subjects practiced the orientation discrimination only in a right oblique standard orientation, which is easier to learn compared to horizontal or vertical orientations. Only one orientation was presented in each trial. The reference orientation at 45° was never presented (Figure 3). Subjects had to decide whether the noise field was tilted clockwise or counterclockwise to the reference orientation by pressing the appropriate arrow key. Auditory feedback was provided. Per daily session we presented 1000 stimuli divided into 10 blocks of 100 stimuli. Subjects were allowed to take a short break between the 10 blocks. We suggested that they closed their eyes for a moment before they continued the task. Each learning session lasted approximately 20 min (corresponding to the tDCS duration).

At the beginning, the orientation of the bars differed by 7° from the reference orientation. After four correct responses, the orientation difference was decreased by 20%, and after a single incorrect response the orientation difference was increased by 20%. To determine the “just noticeable difference” (JND), we used the up-and-down transformed respond-method (UDTR). A positive reversal point was achieved by the turn from right to wrong answers and a negative reversal point from false responses to correct ones. All reversal points were summed up and divided by the number of reversal points, resulting in a geometric mean for each block. The JND was calculated out of the mean values of all 10 blocks for the whole, daily session.

The task setup and procedure were designed based on the approved learning paradigm of Schoups et al. (1995). Each subject had to perform one session on baseline (D1) and on D2, D3, D4 and D5 during tDCS.

### Statistical Analysis

Measurements of PTs and psVEPs were performed on D1 and D5 (two data points). In our data, psVEPs and PT showed low intraindividual variability with high interindividual variability. Levene’s test revealed an inhomogeneity of variances. Hence, we used Student’s paired *t*-test to analyze the tDCS-effect on PT and psVEPs for each group (pre-post-differences). To rule out baseline differences of PT and amplitude ratios between the groups, we used unpaired two-tailed *t*-tests. For these tests, the significance level was adjusted by dividing it by the number of comparisons (0.05/3 = 0.017; Bonferroni correction). The ODT was performed on 5 days. For the analysis of the behavioral data, we used a repeated measurement analysis of variance (ANOVA) with the within-subject factor “time” and between-subject factor “group” in order to find a learning effect over the days, an interaction between learning and groups and group differences. If ANOVA revealed a significant effect, unpaired two-tailed *t*-tests were used for *post hoc* analysis. Before using parametric tests, normal distribution was confirmed by using the Kolmogorov-Smirnov test. In order to show a correlation between excitability parameters and learning parameters, we performed linear bivariate correlation analyses (two-tailed Pearson’s correlation). For all statistical tests, we used the SPSS 21.0 software package (SPSS Software, Munich, Germany).

## Results

### Groups

There were no differences in age and sex between all three groups (univariate ANOVA with age as dependent variable and group and sex as factor; *F*_(1,0.597)_ = 0.446 (sex); *F*_(2,1.291)_ = 0.291 (age)).

### Excitability Parameters

**Figure 4:**
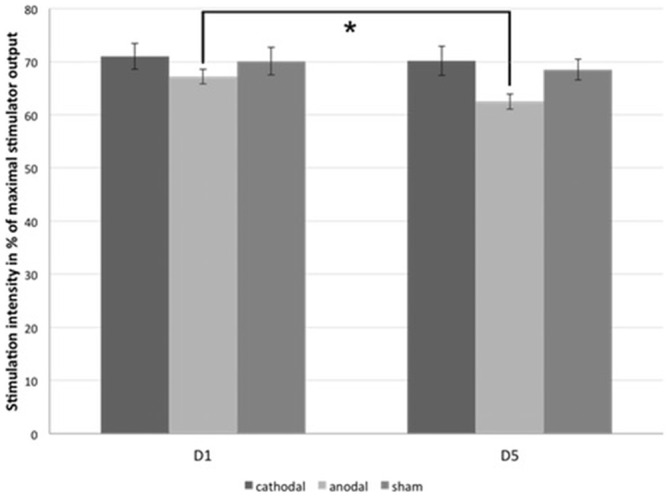
**Phosphene thresholds (PTs).** The figure shows the mean minimal transcranial magnetic stimulation (TMS)-stimulation intensity in percentage of the maximal stimulator output that is able to evoke phosphene perception in subjects of all three tDCS-groups on D1 and D5. *significance level, *p* < 0.017. Error bars indicate standard error of the mean.

**Figure 5:**
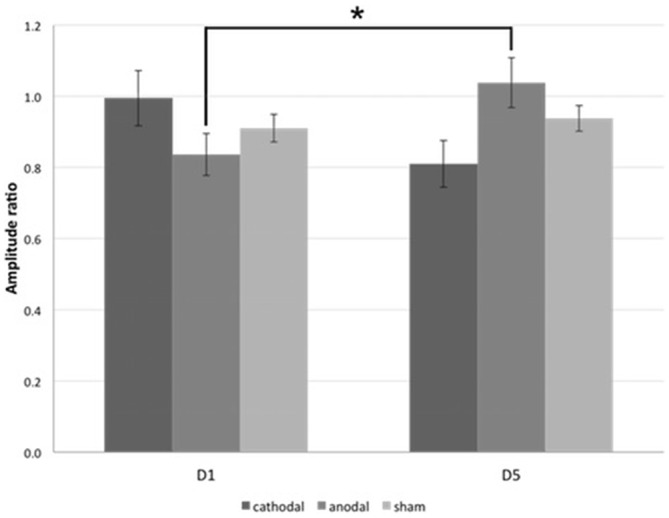
**Paired-stimulation VEP (psVEP).** Mean amplitude ratios of all three groups on D1 and D5 are plotted. *significance level, *p* < 0.017. Error bars indicate standard error of the mean.

Regarding PTs, significant baseline differences were ruled out (anodal vs. cathodal: *p* = 0.190, anodal vs. sham: *p* = 0.343, cathodal vs. sham: *p* = 0.803). Paired two-tailed *t*-tests revealed a significant decrease between D5 and D1 for anodal tDCS group (mean PT_D1_ = 67.2 ± 1.41%, mean PT_D5_ = 62.5 ± 1.44%, *p* = 0.002; Figure 4). There were no significant effects in the cathodal and sham tDCS groups (cathodal tDCS: *p* = 0.608, sham tDCS: *p* = 0.343). For the analysis of paired-stimulation behavior, we calculated the amplitude ratios (A2s/A1) of the cortical evoked responses to paired-pattern-stimulation as mentioned above (Figure 5). We found no differences in amplitude ratio between all three groups in baseline measurements on D1 (anodal vs. cathodal: *p* = 0.122, anodal vs. sham: *p* = 0.310, cathodal vs. sham: *p* = 0.344). When analyzing changes after 4 days of tDCS, we found a significant increase of the amplitude ratio in the anodal tDCS group (mean amplitude ratio D1 = 0.84 ± 0.06, D5 = 1.04 ± 0.07, *p* = 0.003). In the cathodal tDCS group, there was a strong decrease of the amplitude ratio that did not reach the Bonferroni-corrected significance level (mean amplitude ratio D1 = 0.99 ± 0.08, D5 = 0.81 ± 0.07, *p* = 0.039).

### Visual Learning—Orientation Discrimination Task

**Figure 6:**
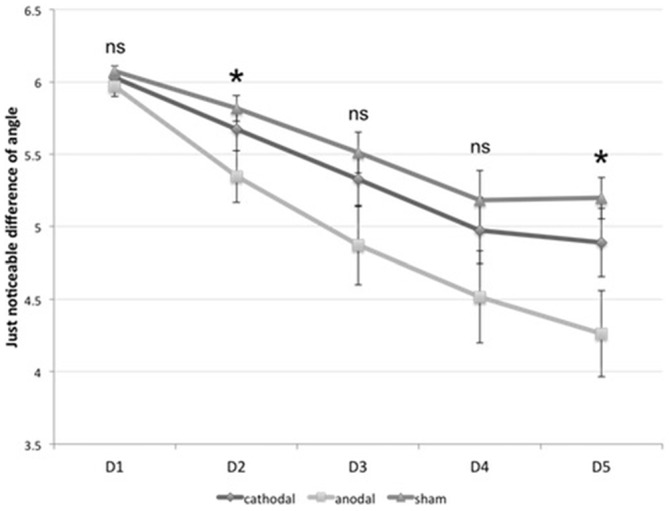
**Visual learning.** The diagram shows the just noticeable difference (JND) in orientation discrimination for all three groups on from D1 to D5. *significance level, *p* < 0.017. Error bars indicate standard error of the mean.

All subjects were able to learn the ODT (within-subject factor “time”, *F*_(4,108)_ = 60.93, *p* < 0.0001). We found a significant interaction between the within-subject factor “time” (*F*_(8,108)_ = 2.904, *p* = 0.006) and the between-subject factor “group” (*F*_(2,27)_ = 3.415, *p* = 0.048). As the *post hoc* *t*-tests revealed, anodal stimulation enhanced the orientation discrimination threshold, whereas cathodal stimulation failed to significantly influence visual learning. There was a significant effect for the anodal group on D2 and D5 in learning orientation discrimination compared to the sham group (D2: *p* = 0.030, D5: *p* < 0.003). We found no significant differences between cathodal and sham stimulation. Figure 6 shows the results.

### Correlation Analysis

**Figure 7:**
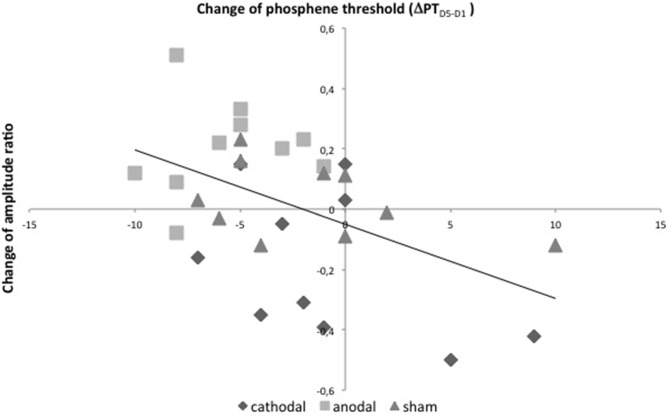
**Correlation analysis.** Linear bivariate correlation analysis of changes of PTs in % of maximal stimulator output between D5 and D1 (*x*-axis) and changes of amplitude-ratios after psVEP between D5 and D1 (*y*-axis) with linear regression. *r* = −0.50, *p* = 0.01, *R^2^* = 0.24.

A correlation analysis was performed between the learning effect ΔJND_D5−D1_ and the change of PT ΔPT_D5−D1_ within the anodal tDCS group. It revealed no significant correlation. Furthermore, the analysis showed a significant negative correlation between both excitability parameters ΔPT_D5−D1_ and Δamplitude ratio_D5−D1_ (*r* = −0.50, *p* = 0.01, *R^2^* = 0.24, Figure 7). No significant correlation was found for the absolute values of amplitude ratio, PT and discrimination threshold.

## Discussion

Two main findings of this study are that anodal tDCS over V1 for four consecutive days is able to improve learning of orientation discrimination compared to cathodal and sham stimulation, and to increase cortical excitability. Despite these significant effects, no significant correlation between excitability and learning parameters within the anodal tDCS group could be found. However, when looking for an overall tDCS effect including data of all 30 subjects, a significant positive correlation between tDCS-induced changes of excitability and visual learning was found. This is the first study testing the efficacy of repeated application of tDCS over V1, demonstrating visual perceptual learning and associated changes of excitability parameters in human subjects.

### Anodal tDCS Decreased TMS-Induced Phosphene Thresholds

In the present study, we demonstrated that anodal tDCS applied on four successive days decreased TMS-induced PT significantly. This result indicates increased excitability in V1. The effect of tDCS on PTs as a marker of excitability in visual cortex has been examined in previous studies (Antal et al., 2003a,b), which demonstrated that tDCS was capable to induce transient alterations of visual cortex excitability. Even if the precise origin of phosphenes is unclear and extrastriate visual-cortical areas (V2, V3) are known to be involved, V1 stimulation alone is capable to modulate phosphene perception (Beckers and Zeki, 1995; Kastner et al., 1998; Cowey and Walsh, 2000; Kammer et al., 2001; Sparing et al., 2002). So far, our results are line with those revealed in previous work.

### Anodal tDCS Reduced Paired-Stimulation Suppression of VEPs

This is the first study using psVEPs to assess the influence of tDCS and visual learning on cortical excitability. In previous studies, our group established a psVEP paradigm in order to obtain an alternative approach to explore excitability of visual cortex (Höffken et al., 2008, 2009). In analogy to paired-stimulation paradigms in the motor and somatosensory system (Kujirai et al., 1993; Klostermann et al., 2000; Schwenkreis et al., 2005; Lenz et al., 2011), psVEPs provide information about paired-stimulation suppression, which is a marker of cortical excitability and is used to characterize plastic changes in V1 (Shagass and Schwartz, 1965; Musselwhite and Jeffreys, 1983; Cantello et al., 2001; Normann et al., 2007; Höffken et al., 2008). We previously reported an enhanced excitability of V1 in patients suffering from migraine (Höffken et al., 2009). In our current study, we demonstrated a significant decrease of paired-stimulation suppression after anodal tDCS in combination with visual learning. Furthermore, we observed no significant increase of paired-stimulation suppression in the cathodal tDCS-group. As previously published by our group, PT and paired-stimulation behavior of VEPs correlate negatively (Höffken et al., 2013), i.e., higher PTs were associated with smaller paired-stimulation ratios. It is conceivable that both methods may be mediated through different underlying mechanisms; both methods target aspects of visual cortex excitability, and reflect a common characteristic of visual cortex. Here, we could partially reproduce our previous findings (Figure 7). We found a significant negative correlation between the change of amplitude ratio and change of PT between D5 and D1 independently of type of stimulation. In contrast to our previous results from 2013, the current study tested interventions in a pre-post design. So, we looked for the stimulation *effects* rather than for the status on D1 or D5. Our current data indicate that not only in the steady state but also after noninvasive cortical intervention both electrophysiological methods reflect common underlying mechanisms. Since tDCS in this study could have altered the function of all neuronal tissue in between Cz and Oz, we have to take parts of the parietal cortex into account, too. Despite much scientific work, the mechanisms mediating paired-stimulation suppression are not fully understood.

### Anodal tDCS Improves Orientation Discrimination

As an important result of our study, we observed that anodal tDCS improves visual perceptual learning (orientation discrimination). Significant effects could be shown on D2 and D5, in comparison with sham-tDCS group. Perceptual learning is characterized by a quite stable and distinct improvement in sensory discrimination after repeated exposure to a particular type of stimulus and is considered as a manifestation of neural plasticity. Neural modifications that occur during perceptual learning are direct evidence of the presence of cortical plasticity in the brain (Gilbert et al., 2001; Li et al., 2004; Carmel and Carrasco, 2008). This fact offers the possibility to modulate cortical activity and perceptual performance via brain stimulation tools like tDCS. Our ODT is a classical visual perceptual learning task that has previously been used by other groups (Matthews et al., 1999; Pirulli et al., 2013). As demonstrated in animal models, it is well known that specialized V1-neurons are involved in orientation discrimination performance (Schoups et al., 2001). Significant differences between anodal tDCS and sham tDCS could be observed at D2 and D5, but not on D3 and D4. Next to our study, only one more publication applied tDCS over more than 1 day: Reis et al. (2009) investigated tDCS effects on motor learning and consolidation, stimulating M1 over a period 5 days. With regard to the learning curve, Reis and colleagues demonstrated the largest difference on the last day (D5). However, statistical differences on D2–D4 were not explicitly mentioned. To our best knowledge, there are no further studies using a 5-day-tDCS-protocol on human learning. In our study, the early effect on D2 might be explained by the idea of strengthening preexisting synapses by tDCS (short-term plasticity). These effects might become saturated early on D3. Furthermore, we have to take attention deficits into account. Other plastic neuronal effects leading to LTP mechanisms might be initiated by repeated applications of tDCS and learning itself and become visible on later days (D5). But, so far, there are few experiments focusing on effects of noninvasive brain stimulation and neuronal plasticity of repeated stimulation over several days. Our study is the first one to investigate such effects in the visual system. Further studies are needed to clarify underlying neuronal mechanisms. Basically, in all tDCS studies, one has to consider that reported stimulation effects might be influenced by inadvertent stimulation of nearby cortices. Generally, tDCS studies use 5 cm × 7 cm rubber electrodes that do not allow for stimulation of small cortical areas, unlike a figure-of-eight TMS coil. With regard to cortical areas involved in orientation discrimination, so far there are no studies that directly investigated the role of other visual cortices (Schoups et al., 2001). Based on Schoups’ results, we do not expect a relevant effect on other visual cortices, but we cannot rule it out completely.

Recently, Fertonani and coworkers showed that noninvasive electrical brain stimulation is capable of improving visual perceptual learning (orientation discrimination; Fertonani et al., 2011). A single session of high-frequency transcranial random-noise stimulation (tRNS) of V1 led to significantly improved performance accuracy. Moreover, it was demonstrated that cathodal, anodal and sham tDCS had no effect. Interestingly, Fertonani et al. proposed that tDCS had no effect because the constant electrical field would allow the membrane responses to adapt and return to an initial “resting” state.

Our findings are contradictory to this result. Two major differences between the study designs may contribute to an explanation of the differences: first, we applied tDCS successively on 4 days and not only on one single day. This prolonged application presumably led to the significant learning effect in anodal tDCS group on D5. In another study using repetitive stimulation on several days, Fritsch et al. (2010) and Reis et al. (2009) also applied tDCS over M1 for five consecutive days and measured motor skill parameters in order to evaluate motor consolidation. They demonstrated that repetitive anodal stimulation resulted in enhanced motor learning while observing no effect on D1. In addition, they showed on molecular level that improvement of motor learning required repetitive low-frequency synaptic activation and activity-dependent BDNF secretion, indicating long-term potentiation mechanisms, which might explain why significant effects were not observed after a single tDCS session but after five sessions.

Secondly, before the first tDCS session was applied on D2 in our study, all subjects had already learned orientation discrimination for 20 min on D1 (baseline measurements). Therefore, we suppose that orientation-sensitive specialized V1-neurons were primed and, hence, caused lower detection thresholds before the first tDCS session started. Due to this, they may have been more susceptible for the anodal tDCS influence. Metaplastic mechanisms like gating might play a role in this case (Ziemann and Siebner, 2008).

A further minor difference is that both studies used different parameters to evaluate the learning effect. We used JND, which describes an angle. (Fertonani et al., 2011) analyzed the average orientation sensitivity by calculating the d’value.

### Changes of Cortical Excitability in Anodal tDCS group do not Correlate with Changes of Orientation Discrimination

Studies showed that excitability levels in cortical areas can correlate with learning improvement, which was proved for motor and tactile learning. Thus, in M1 excitability has been suggested as a marker of learning and use-dependent plasticity. Motor training is able to increase cortical excitability (Cirillo et al., 2010, 2011), while increased motor cortical excitability positively correlates with performance improvements in simple motor tasks (Muellbacher et al., 2001). However, some studies on motor learning were unable to replicate this correlation. Factors like type and complexity of task seem to be relevant. For example, Lissek et al. (2014) using a more complex motor task found no changes of cortical excitability after task training and performance. Vice versa, some motor studies using serial reaction time tasks demonstrated no learning effects but increased cortical excitability. These results indicate that more than one cortical area needs to be involved and is required for motor learning. A neuronal network consisting of basal ganglia, supplemental motor cortex, premotor cortex and cerebellum has been described as being involved in motor learning (Mima et al., 1999; Ungerleider et al., 2002). Furthermore, in the somatosensory system, (Pleger et al., 2001) demonstrated that the baseline excitation level predicts the learning effect of a 2-point-discrimination task. In our study, we did not observe such a link for the anodal tDCS group, despite the fact that we found significant effects for learning and cortical excitability, respectively, in this group. Most likely, this lack of correlation might be due to the low number of subjects (*n* = 10) and the inhomogeneity of data. Moreover, similar to the motor system, it cannot be ruled out that other brain regions might be involved in this type of visual learning. Our study did not investigate this aspect. Upcoming studies should involve magnetic resonance imaging to answer this question.

### Different Effects of Anodal and Cathodal tDCS

The lack of a significant effect of cathodal tDCS requires some further explanation. Considering previous work, there are some studies that showed effects of tDCS limited to one polarity of stimulation in different modalities (motor, somatosensory, visual system; Nitsche and Paulus, 2000; Antal et al., 2001; Baudewig et al., 2001; Priori, 2003; Matsunaga et al., 2004). So, the present results are not unique. Apparently, different parameters influence the stimulation effects, such as morphology, type, and orientation of cortical neurons relative to applied electrical fields, as well as background level of activity, type of task, task characteristics and further more (Matsunaga et al., 2004; Miniussi et al., 2008; Radman et al., 2009). In our current study, we observed significant reduction of paired-pulse suppression after 5 days of anodal tDCS over V1. After 5 days of cathodal tDCS, we saw a clear trend to increased paired-pulse suppression, which, however did not reach statistical significance, probably due to the low number of subjects. For PT, again, anodal tDCS showed a clear statistical significant effect. Here, cathodal tDCS showed absolutely no effect, no trend and no significant difference. These results show that even after 5-day-application different effects of anodal and cathodal tDCS on V1 could be demonstrated. Which factors determined these findings remains totally unclear. Further experiments are required to address this point.

## Conclusion

In conclusion, this work highlights the effects of anodal tDCS in modulating excitability and visual, perceptual learning. Our data support the idea of using tDCS in a repeated, daily manner on consecutive days to induce more stable after-effects. However, this study also indicates that further work has to be done to determine optimal stimulation parameters such as polarity in different neuronal systems and different stimulation timing and duration. Furthermore, the neuronal mechanisms underlying the observed effects should be investigated via neuromodulation and/or multimodal neuroimaging techniques.

## Author Contributions

MS-K designed the study, acquired, analyzed and interpreted the data, and drafted the article. KB acquired, analyzed and interpreted the data, and drafted the article. OH, HRD, PS and MT designed the study, interpreted data, and revised the manuscript.

## Conflict of Interest Statement

The authors declare that the research was conducted in the absence of any commercial or financial relationships that could be construed as a potential conflict of interest.

## Abbreviations

V1: primary visual cortex
M1: primary motor cortex
VEP: visually evoked potentials
psVEP: paired- stimulation visually evoked potentials
ODT: orientation discrimination task
PT: phosphene threshold
mA: milliampere
tDCS: transcranial direct current stimulation
tRNS: transcranial random noise stimulation
TMS: transcranial magnetic stimulation
S1: primary somatosensory cortex
D1: day 1
D5: day 5
JND: just noticeable difference.
